# Differential aging of growth plate cartilage underlies differences in bone length and thus helps determine skeletal proportions

**DOI:** 10.1371/journal.pbio.2005263

**Published:** 2018-07-23

**Authors:** Julian C. Lui, Youn Hee Jee, Presley Garrison, James R. Iben, Shanna Yue, Michal Ad, Quang Nguyen, Bijal Kikani, Yoshiyuki Wakabayashi, Jeffrey Baron

**Affiliations:** 1 Section on Growth and Development, Eunice Kennedy Shriver National Institute of Child Health and Human Development, National Institutes of Health, Bethesda, Maryland, United States of America; 2 Molecular Genomics Core, Office of the Scientific Director, Eunice Kennedy Shriver National Institute of Child Health and Human Development, National Institutes of Health, Bethesda, Maryland, United States of America; 3 DNA Sequencing and Genomics Core, National Heart, Lung, and Blood Institute, National Institutes of Health, Bethesda, Maryland, United States of America; Lincolns Inn Fields Laboratory, United Kingdom of Great Britain and Northern Ireland

## Abstract

Bones at different anatomical locations vary dramatically in size. For example, human femurs are 20-fold longer than the phalanges in the fingers and toes. The mechanisms responsible for these size differences are poorly understood. Bone elongation occurs at the growth plates and advances rapidly in early life but then progressively slows due to a developmental program termed “growth plate senescence.” This developmental program includes declines in cell proliferation and hypertrophy, depletion of cells in all growth plate zones, and extensive underlying changes in the expression of growth-regulating genes. Here, we show evidence that these functional, structural, and molecular senescent changes occur earlier in the growth plates of smaller bones (metacarpals, phalanges) than in the growth plates of larger bones (femurs, tibias) and that this differential aging contributes to the disparities in bone length. We also show evidence that the molecular mechanisms that underlie the differential aging between different bones involve modulation of critical paracrine regulatory pathways, including insulin-like growth factor (Igf), bone morphogenetic protein (Bmp), and Wingless and Int-1 (Wnt) signaling. Taken together, the findings reveal that the striking disparities in the lengths of different bones, which characterize normal mammalian skeletal proportions, is achieved in part by modulating the progression of growth plate senescence.

## Author summary

The various bones found in human extremities vary dramatically in size. For example, the femur in the thigh is approximately 20 times longer than the phalanges of the toes. The mechanisms that regulate how much a particular bone grows to attain the skeletal proportions of each animal remain mostly unknown. In this study, we sought to uncover these mechanisms and found that this scaling in skeletal size is achieved in part by modulating the rate of aging of growth plates in different bones. Bone elongation occurs at the cartilaginous growth plates and proceeds rapidly in early life but slows and eventually stops due to a developmental program termed “growth plate senescence,” which is used to describe the gradual decline in growth plate function with age. We discovered that this developmental program is more advanced in shorter bones than in longer bones and that this differential aging contributes to the disparities in bone growth. We show that the molecular mechanisms that underlie this differential aging between bones involve differential expression of specific local signaling pathways, which regulate cell proliferation and differentiation in the growth plate. Taken together, we conclude that the striking disparities in the lengths of different bones—characteristic of normal mammalian skeletal proportions—are achieved in part by modulating the progression of aging in the growth plates.

## Introduction

Bone elongation is driven by endochondral ossification that takes place at the growth plate, a cartilaginous structure found near the ends of long bones [1,2]. Each growth plate is composed of 3 histologically distinct zones. The resting zone, which lies closest to the end of the bones, consists of small, round progenitor chondrocytes with a finite capacity to self-renew [3]. These cells give rise to columnar clones of flat, rapidly proliferating chondrocytes in the proliferative zone. Proliferative chondrocytes undergo terminal differentiation into hypertrophic zone chondrocytes, which reside in the region nearest to the center of the bone. Hypertrophic chondrocytes either transdifferentiate into bone-forming osteoblasts [4] or undergo apoptosis, leaving a cartilage matrix scaffold that is remodeled into bone by invading osteoblasts and osteoclasts. The rate of long bone elongation (length/time) is primarily determined by the rate of chondrocyte proliferation (cells/time) per column multiplied by the cell height (length/cell) achieved after chondrocyte hypertrophy [5,6]. During infancy, the growth plate functions robustly, causing rapid bone lengthening. However, with age, bone growth gradually slows and eventually ceases due to a developmental program termed “growth plate senescence,” which involves a progressive decline in growth plate function and extensive structural and molecular changes in the growth plate [7].

During mammalian embryonic development, all long bones form from mesenchymal condensations of similar size [8,9]. However, different long bones diverge in growth rate, ultimately leading to dramatic differences in bone length. For example, adult human femurs are more than 10 times longer than the phalanges of the fingers and toes [10]. In the current study, we sought to explore the mechanisms that underlie the dramatic differences in growth rates and lengths of different long bones by comparing growth plates in shorter bones (metacarpals and proximal phalanges) versus growth plates of longer bones (distal femurs and proximal tibias) in 2 species, the mouse and the rat, at the cellular and molecular levels.

## Results

**Fig 1:**
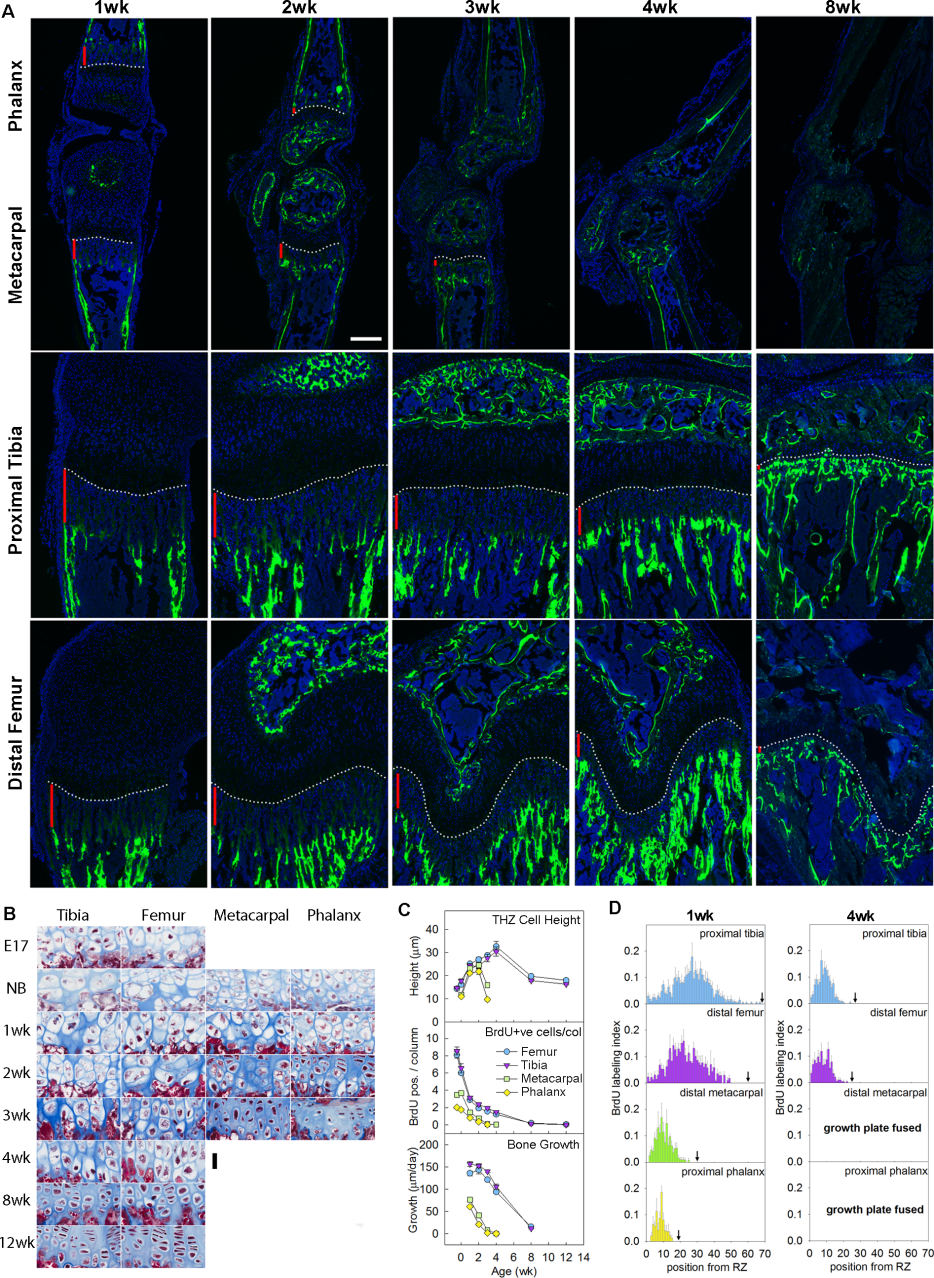
Disparities in chondrocyte proliferation, chondrocyte hypertrophy, and bone growth rate between shorter and longer bones. (A) Fluorescent images of proximal tibias, distal femurs, distal metacarpals, and proximal forelimb phalanges from mice at various postnatal ages. Rate of longitudinal bone growth was determined from the distance (vertical red bars) between the chondro-osseous junction (white dotted line) and the calcein-labeled (fluorescent green) bone. DAPI was used for counterstain. Scale bar, 200 μm. (B) Masson Trichrome–stained histological sections of hypertrophic zone of proximal tibias, distal femurs, distal metacarpals, and proximal forelimb phalanges, from C57BL/6 mice at E17.5 and various postnatal ages. Hypertrophic cell height diminished earlier in the metacarpals and phalanges. Scale bar, 30 μm. (C) Quantitative histological measurements of TH cell height (upper panel) and number of BrdU-labeled cells per column in the proliferative zone (middle panel), and rate of bone growth measured by calcein-labeling (lower panel). (D) Position-specific BrdU labeling indices of proliferative zone of proximal tibias, distal femurs, distal metacarpals, and proximal forelimb phalanges in 1- and 4-week-old mice. Cell position 1 denotes the proliferative chondrocyte closest to the resting zone, and black arrow indicates the cell position where the proliferative zone ends and the pre-hypertrophic region starts. Position-specific BrdU labeling indices at other time points are depicted in S4 Fig. Raw values for Fig 1C and 1D are available in S1 Data. BrdU, 5-bromo-2-deoxyuridine; E17.5, embryonic day 17.5; TH, terminal hypertrophic.

As expected, the rates of bone elongation at the proximal tibia and the distal femur, measured by calcein labeling, were greater than those of the metacarpal bones and proximal phalanges (Fig 1A and 1D). Some previous studies have attributed these differences in growth rate between bones to differences in the size attained by the hypertrophic chondrocytes of the growth plate [11,12]. However, the rate of bone elongation is also dependent on chondrocyte proliferation and is approximated by the height of the terminal hypertrophic chondrocyte in the column multiplied by the chondrocyte proliferation rate per cell column [5,6]. We therefore asked whether differences in proliferation rates between bones might also contribute to differences in growth rate. We found that, in mouse, both the height of the terminal hypertrophic cell (Fig 1B and 1C, upper panel) and the proliferation rate per column (Fig 1C, middle panel and S1 Fig) were diminished in the metacarpal and proximal phalangeal growth plates compared with the proximal tibial and distal femoral growth plates, and the disparities in the proliferation rate were greater than the disparities in cell size (Fig 1C and S1 Table). For example, in the 1-week mouse, the metacarpal bone growth rate was 49% of the tibial bone growth rate, and the proliferation rate per column in the metacarpal growth plate was 46% that of the tibial growth plate (see S1 Table). In contrast, there was little difference in hypertrophic cell size between the 2 bones at that same time point. A similar pattern was found in the rat (S2 Fig, S2 Table). Thus, the proliferation rate per column appears to be the dominant factor involved. Although the proliferation rate per column differed markedly between bones, the proliferation rates per cell (5-bromo-2-deoxyuridine [BrdU] labeling index) were similar (S3 Fig). We next analyzed the BrdU labeling index at each position along the chondrocyte columns (proliferation profile). We found that, in the tibial and femoral growth plates, proliferation extended farther down the columns compared with the metacarpals and phalanges (Fig 1D and S4 Fig). These findings suggest that a chondrocyte near the top of the growth plate in the larger bones would go through more rounds of cell division before slowing and ceasing proliferation compared with the smaller bones.

**Fig 2:**
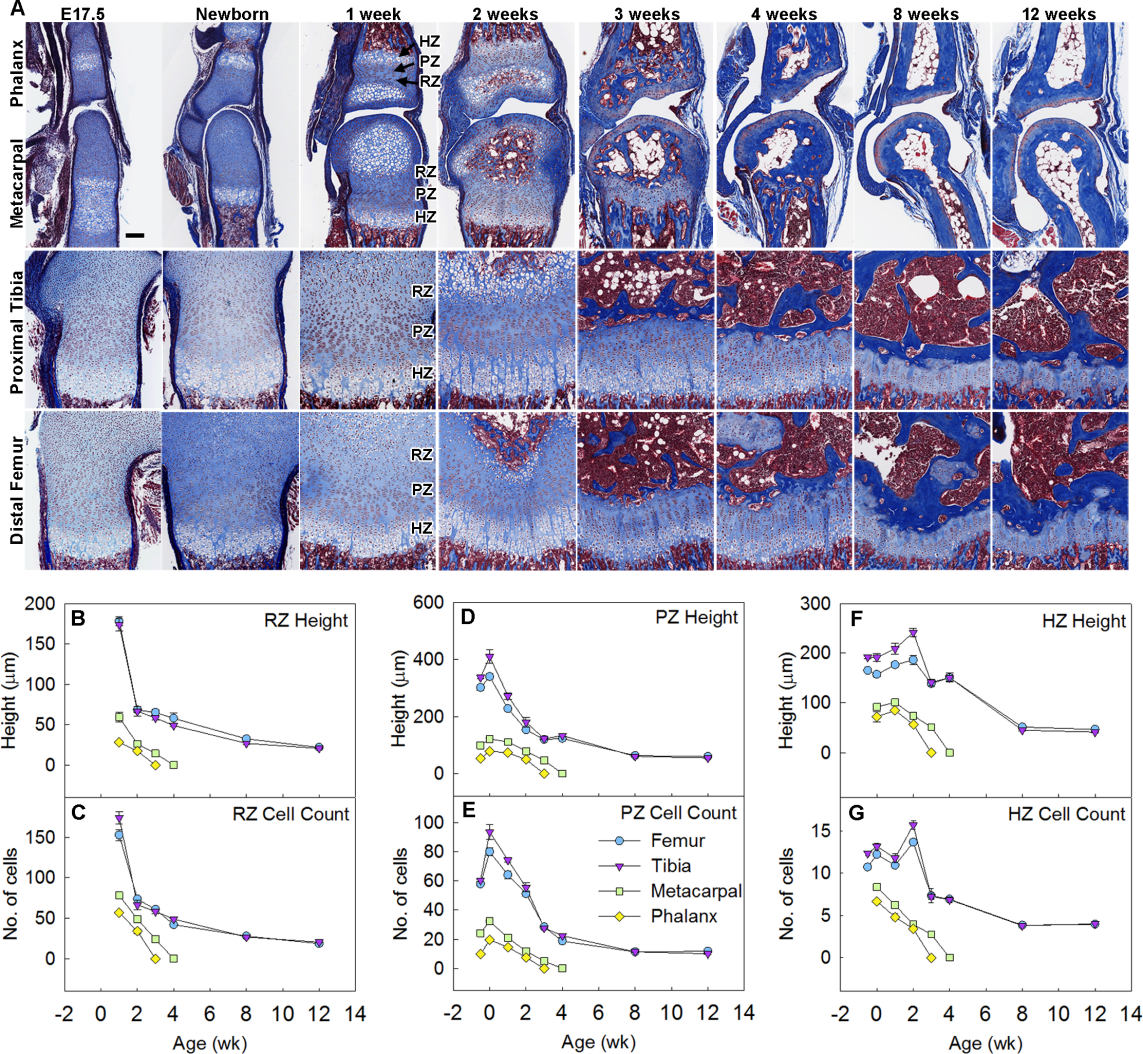
Growth plate senescence is more advanced in shorter bones than in longer bones. (A) Masson Trichrome–stained histological sections of proximal tibias, distal femurs, distal metacarpals, and proximal forelimb phalanges from C57BL/6 mice at various ages. Cartilage matrix stains light blue; bone matrix, dark blue. Epiphyseal fusion (disappearance of growth plate) occurs at approximately 3 weeks in phalanges and 4 weeks in metacarpals but has not yet occurred at 12 weeks in tibias or femurs. Scale bar, 100 μm. (B–G) Quantitative histological measurements of RZ height (panel B) and cell count (panel C), PZ height (panel D) and cell count per column (panel E), HZ height (panel F) and cell count per column (panel G), in each of the 4 growth plates at various ages. *N* = 6, mean ± SEM. Raw values for Fig 2B–2G are available in S1 Data. HZ, hypertrophic zone; PZ, proliferative zone; RZ, resting zone.

We noticed that the time course of the bone growth rate (Fig 1C, bottom panel), the proliferation rate per column (Fig 1C, middle panel), and the proliferation profile (Fig 1D and S4 Fig) in smaller bones approximately paralleled the time course in the larger bones but appeared to be either left-shifted and/or down-shifted. We hypothesized that a left-shift may have occurred because the developmental program of growth plate senescence is more advanced in shorter bones. Growth plate senescence is characterized not only by a decline in proliferation rates but also by a gradual structural involution of the growth plate, including declines in the overall height of each growth plate zone and the number of chondrocytes in each zone. Consistent with our hypothesis, all of these senescent changes were more advanced in the metacarpals and the proximal phalanges compared with the distal femurs and proximal tibias (Fig 2A), such that the time courses for the shorter bones were shifted to the left (Fig 2B–2G, S4 Fig). Prior studies indicate that, when the growth rate of the growth plate approaches 0, the cartilaginous growth plate is replaced by bone [13]. This final stage of growth plate senescence—termed “epiphyseal fusion”—occurred earlier in the shorter bones (2–3 weeks of age) than in the longer bones (unfused at 12 weeks, the last time point studied) (Fig 2A), further confirming that the overall senescence program occurs earlier in shorter bones. We similarly analyzed skeletal maturation in the rat and found that all functional and structural makers of growth plate senescence studied were more advanced in metacarpals and phalanges than in tibias and femurs (S2 Fig) and that fusion occurred earlier in the shorter bones. Taken together, these observations demonstrate that growth plate senescence is more advanced in the shorter bones, thus explaining the slower growth rate and diminished length.

Growth plate senescence is associated with an underlying genetic program in chondrocytes involving extensive age-dependent changes in gene expression [7]. We therefore hypothesized that this senescence-associated genetic program would be more advanced in the shorter bones. To test this hypothesis, we used laser capture microdissection (LCM) to separately isolate the proliferative and hypertrophic zones of mouse and rat growth plates and used RNA sequencing (RNA-Seq) to compare gene expression profiles in the proximal tibial growth plate at 1 and 4 weeks of age and the proximal phalangeal growth plate at 1 week of age. We first examined the expression levels of genes previously reported as markers for the proliferative and hypertrophic zones [7]. These expression patterns confirmed the spatial accuracy of our dissection (S5 Fig).

**Fig 3:**
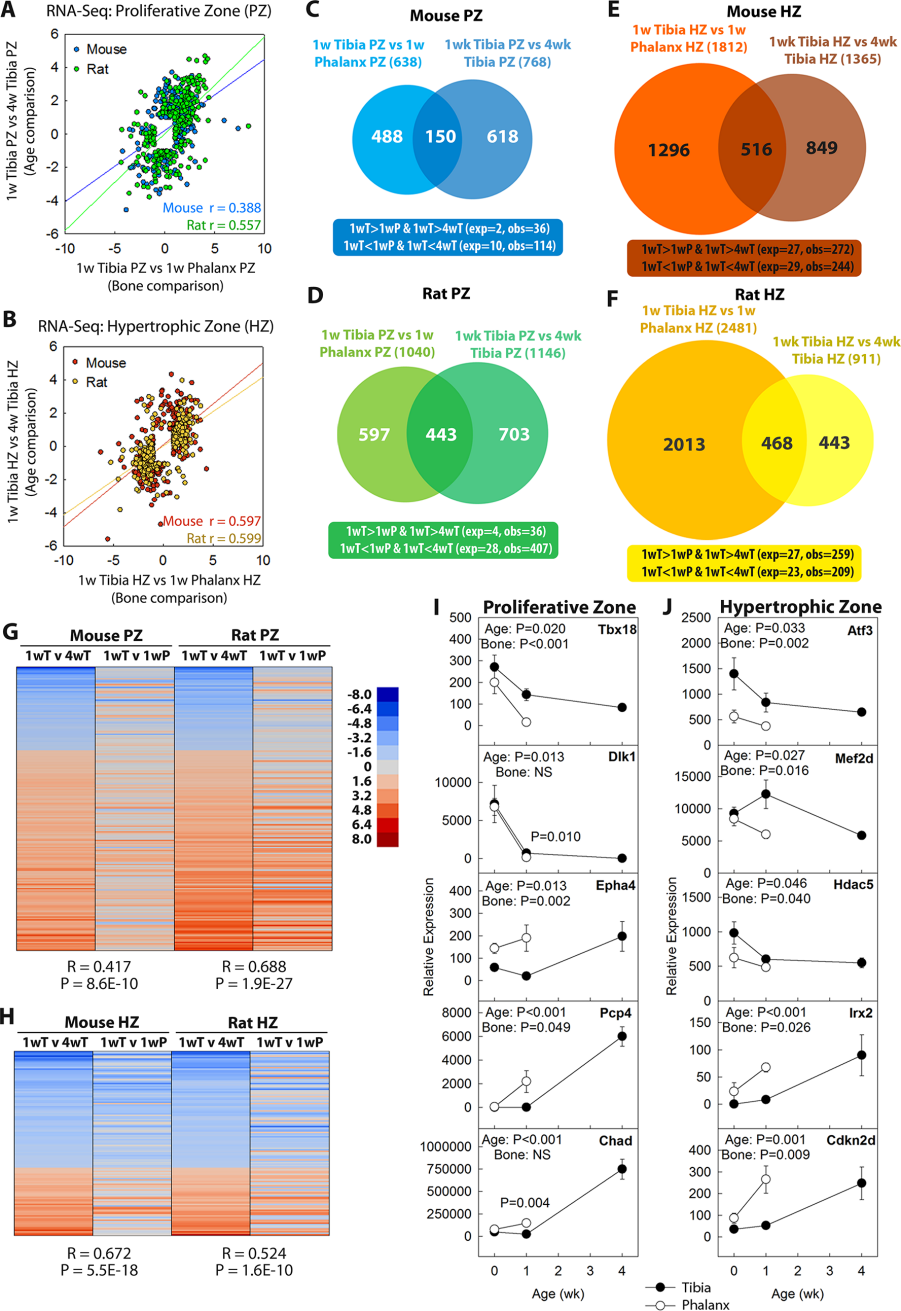
Growth plate senescence-associated changes in gene expression are more advanced in the phalangeal growth plates than in tibial growth plates. RNA-Seq was performed on laser capture micodissected PZ or HZ of mouse or rat proximal tibias (at age 1 and 4 weeks) or proximal phalanges (at 1 week). (A, B) Positive correlation (mouse PZ, *p* < 1 × 10^−44^; mouse HZ, *p* < 1 × 10^−139^; rat PZ, *p* < 1 × 10^−254^; rat HZ, *p* < 1 × 10^−281^; Pearson’s correlation) between senescent changes (log_2_[fold differences]) in tibial growth plate (1 week versus 4 weeks, >4-fold; FDR < 0.05) and differential expression between bones at 1 week (tibia versus phalanx >4-fold; FDR < 0.05), in PZ (panel A) and HZ (panel B) of both species. (C–F) Venn diagram depicting numbers of genes with significant changes (>4-fold; FDR < 0.05) in expression between 1 and 4 weeks of age in tibial growth plates and/or significant differences between tibia and phalanx at 1 week of age. Overlaps were greater than expected by chance (chi-squared, *p* < 0.0001) in both zones of mouse (panel C and E) and rat (panel D and F). (G, H) Heatmap of genes showing senescence-associated changes in expression (1 versus 4 weeks in tibia; >2-fold, FDR < 0.05, in both species; 200 genes in PZ, 129 genes in HZ). The senescence-associated changes correlated with differential expression between 1-week phalanx and tibia, suggesting that senescence-associated changes are more advanced in phalanges than tibias (*R*, Pearson’s correlation coefficient). Scale bar represents log_2_(fold differences). (I, J) qPCR in a subset of genes showed that changes in gene expression (mRNA normalized to 18S RNA) began before 1 week of age and confirmed that changes in the phalanges tended to be more advanced than in tibias. *p*-Values for age and type of bone. Raw values are available in S1 Data. exp, number of overlapping genes expected by chance; FDR, false discovery rate; HZ hypertrophic zone; NS, not significant; obs, number of observed overlapping genes; P, phalanx; PZ, proliferative zone; qPCR, quantitative PCR; RNA-Seq, RNA sequencing; T, tibia; w, week.

If age-dependent changes in gene expression were more advanced in the phalanges, then for genes that showed decreasing expression with age (1 versus 4 weeks) in the tibia, the expression would be lower in 1-week phalanges than in 1-week tibias (S6 Fig). Conversely, for genes that showed increasing expression with age (1 versus 4 weeks) in tibia, the expression would be higher in 1-week phalanges than in 1-week tibias (S6 Fig). Therefore, one would expect a correlation between gene expression changes with age and gene expression differences between bones (S6 Fig). Consistent with this prediction, for age-regulated genes (>4-fold; false discovery rate [FDR] < 0.05), we found strong positive correlations between the age comparison in tibia (1 versus 4 weeks) and the bone comparison at 1 week (tibia versus phalanx). This correlation was found in both proliferative and hypertrophic zones of both mouse and rat (Fig 3A and 3B, all *p* < 0.0001). Similarly, there was a much greater than expected (*p* < 0.0001, chi-squared test) overlap between genes significantly (>4-fold; FDR < 0.05) down-regulated with age in the tibia, and genes with significantly lower expression in the phalanx than in the tibia at 1 week. A similar pattern was seen for genes up-regulated with age (Fig 3C–3F). To test our hypothesis further, we focused on evolutionarily conserved components of the program by generating a list of genes significantly up- or down-regulated with age in both mouse and rat (>2-fold; FDR < 0.05, see S3 Table and S4 Table). Again we observed a strong tendency for genes that were down-regulated with age to show lower levels of expression in 1-week phalanges than in 1-week tibias, and we observed the converse pattern for genes up-regulated with age (Fig 3G and 3H, Pearson’s correlation, all *p* < 0.0001). Quantitative PCR (qPCR) confirmed that age-dependent changes in gene expression occurred earlier in the phalanx than in the tibia and extended these observations to an earlier time point (0 weeks) (Fig 3I and 3J). Thus, the developmental genetic program associated with senescence is left-shifted in time in the shorter bones compared with the longer bones. Collectively, our findings suggest that growth plate senescence is more advanced in shorter bones in terms of structural, functional, and molecular components of this chondrocytic developmental program.

**Fig 4:**
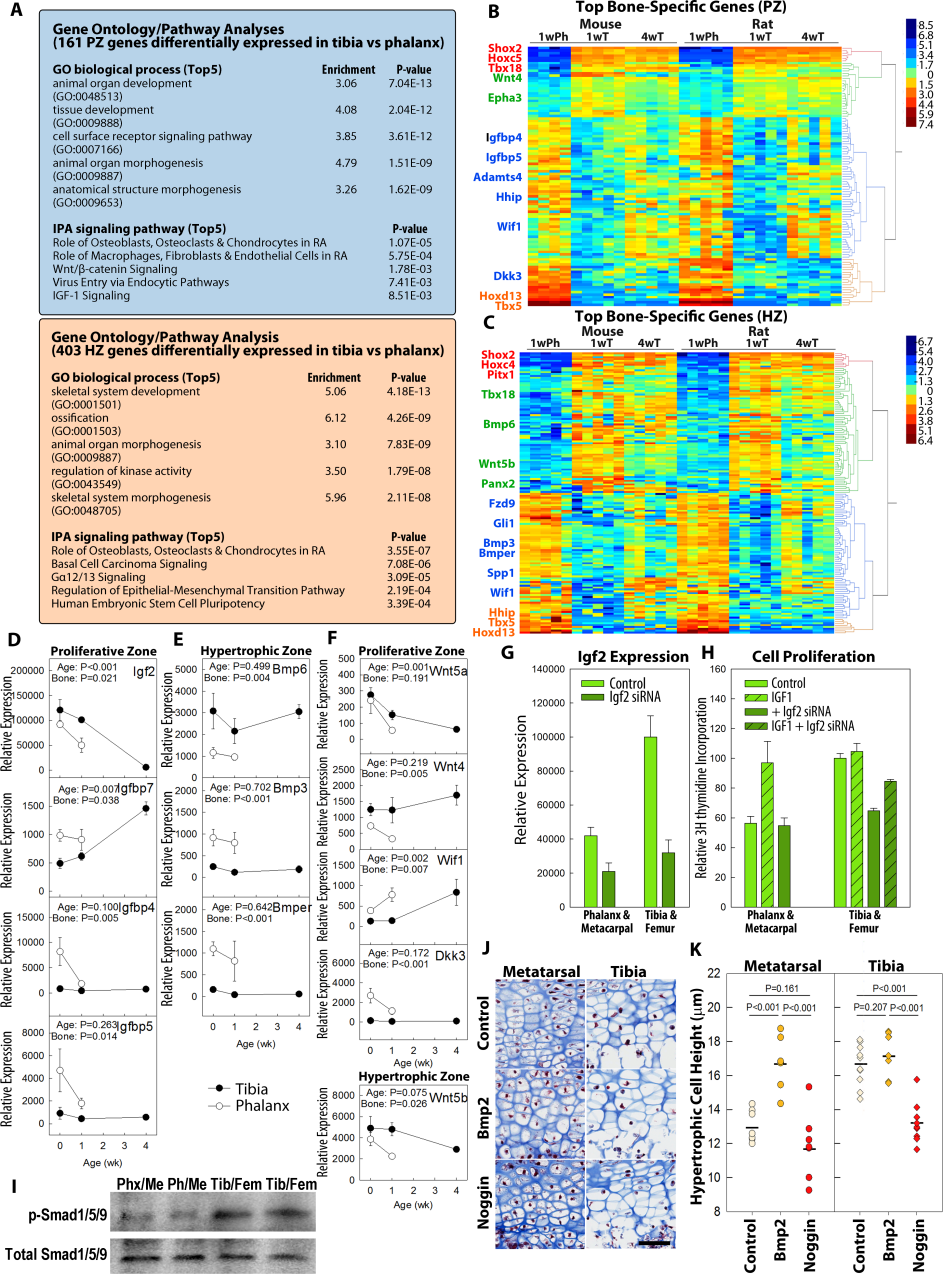
Differences in specific paracrine signaling pathways contribute to disparities in growth plate function between different bones. RNA-Seq was used to identify genes differentially expressed between proximal tibia and proximal phalanx (>2-fold, FDR < 0.05, both species, age 1 week). (A) Gene ontology and signaling pathway analyses showed enrichment for developmental-related functions and identified pathways previously implicated in growth plate biology. (B, C) Heatmaps were generated by hierarchical clustering of the 150 genes that showed the greatest differential expression between tibia and phalanx. Scale bar represents log_2_ (fold differences). (D–F) Specific genes from IGF (panel D), BMP (panel E), and Wnt (panel F) signaling pathways that showed significant differential expression between 1-week tibia and phalanx by RNA-Seq were selected for validation by qPCR and to determine time course. (G, H) When cultured in monolayer, primary mouse chondrocytes isolated from tibias showed higher Igf2 expression (panel G, light green bars) and more rapid proliferation (panel H, open light green bars) than those from phalanges. Proliferation of phalangeal chondrocytes, but not tibial chondrocytes, responded positively to exogenous Igf1 (panel H, striped light green bars). When treated with siRNA against Igf2 (no exogenous Igf1; panel G, dark green bars), proliferation was inhibited (versus control) in tibial chondrocytes but not phalangeal chondrocytes (panel H, open dark green bars). This inhibition was partially reversed by exogenous Igf1 (panel H, striped dark green bar). (I) Western blot showed higher levels of p-SMAD1/5/9 in tibial/femoral growth plate chondrocytes than metacarpal/phalangeal chondrocytes, implying more active BMP signaling in the longer bones. (J, K) Neonatal mouse tibias and metatarsals were treated with Bmp2 or Noggin in culture for 3 days, followed by histological examination for chondrocyte hypertrophy. Scale bar, 50 μm. *N* = 8–10; horizontal line represents sample means. Raw values for Fig 4D–4H, and K are available in S1 Data. BMP, bone morphogenetic protein; FDR, false discovery rate; IGF, insulin-like growth factor; pSMAD1/5/9, phosphorylated SMAD1/5/9; qPCR, quantitative PCR; RNA-Seq, RNA sequencing; siRNA, small interfering RNA; Wnt, Wingless and Int-1.

We next investigated the molecular pathways underlying the observed differences between bones in the progression of growth plate senescence. In addition to the age-dependent differences in gene expression between bones described above, we speculated that there may also be age-independent differences in gene expression that differentially modulate important signaling pathways in different bones. We therefore generated a list of genes (S5 Table and S6 Table) that were differentially expressed in the 1-week proximal tibia versus the phalanx, in both species (>2-fold; FDR < 0.05). Gene ontology analysis of these bone-specific genes showed a general enrichment of skeletal-related and developmental functions (Fig 4A, S7 Table and S8 Table). Pathway analysis identified several signaling pathways implicated in chondrocyte function, such as Wingless and Int-1 (Wnt) signaling and insulin-like growth factor (Igf) signaling in the proliferative zone as well as basal cell carcinoma signaling (involving hedgehog signaling) in the hypertrophic zone (Fig 4A, S9 Table and S10 Table). We visualized the most significantly differentially expressed genes in either the proliferative zone or the hypertrophic zone using heatmaps (Fig 4B and 4C) and showed that, for some of these differentially expressed genes (such as *Shox*, *Tbx5*, and *Hoxd13*), the expression in 1-week phalanges differs from both the 1-week and 4-week tibias, suggesting age-independent differential expression; on the other hand, for other genes (such as *Adamts4* and *Epha3* in proliferative zone and *Panx2* and *Fzd9* in hypertrophic zone), the expression in 1-week phalanges differed from 1-week tibias but resembled the 4-week tibias, suggesting that the difference in expression between phalanges and tibias at 1 week originates from more advanced senescence in the phalanges. We noticed that many of the antagonists or inhibitors of these paracrine signaling pathways, such as *Igfbp4*, *Igfbp5*, *Dkk3*, *Wif1*, and *Bmp3*, were expressed at higher levels in the phalangeal growth plates, while agonists, such as *Bmp6*, *Wnt4*, and *Wnt5b*, were expressed at higher levels in the tibial growth plates (Fig 4B and 4C).

Based on these observations, we hypothesized that some components of key paracrine signaling pathways may be differentially expressed between different bones leading to differing growth rates. Our bioinformatic analysis implicated many signaling pathways, but we chose to focus on specific pathways that were previously known to regulate skeletal growth. We first investigated Igf signaling, which is a critical regulator of growth plate chondrocyte proliferation [14] and hypertrophy [15] (S7 Fig, top panel). Consistent with the RNA-Seq results, we confirmed that 2 Igf-binding proteins (Igfbp)—Igfbp4 and 5—which negatively regulate the local availability of ligands to the Igf receptor and thus inhibit Igf signaling [16], were more highly expressed in the proliferative zone of the phalanges compared with tibias (Fig 4D). A third Igfbp, *Igfbp7*, which we identified as a senescence-related gene previously [17] and also in the current study, was up-regulated with age in the tibia and showed higher expression in the phalanges at birth and at 1 week of age (Fig 4D). In contrast, *Igf2*, which was previously found to decline with age in the growth plate [17], showed an earlier decline in the proliferative zone of the phalanx (Fig 4D). To test for a causal relationship between a difference in Igf signaling and the difference in chondrocyte proliferation, we next cultured murine growth plate chondrocytes isolated from proximal tibias and distal femurs or from phalanges and metacarpals of 1-week-old mice. The chondrocytes from the combined tibias/femurs exhibited higher levels of *Igf2* expression (Fig 4G) and more robust proliferation (Fig 4H) than chondrocytes from the phalanges/metacarpals. Addition of Igf1 to the culture medium increased proliferation of chondrocytes isolated from phalanges/metacarpals to match that of untreated chondrocytes from the tibias/femurs. In contrast, addition of Igf1 to the chondrocytes from tibias/femurs did not stimulate additional proliferation. These results suggest that, in phalangeal/metacarpal chondrocytes, lower Igf signaling limits proliferation, whereas in tibial/femoral chondrocytes, Igf signaling is already optimal for proliferation. We next hypothesized that this apparent difference in Igf signaling was due in part to the greater Igf2 expression in tibial/femoral chondrocytes. Consistent with that hypothesis, suppression of endogenous Igf2 expression with siRNA (Fig 4G) suppressed proliferation in tibial/femoral chondrocytes but not phalangeal/metacarpal chondrocytes (Fig 4H). Lastly, when Igf1 was added back to these siRNA-treated tibial/femoral chondrocytes, they now resembled chondrocytes from the phalanges/metacarpals in that they responded positively to exogenous Igf1 (Fig 4H). These data together strongly suggest that tibial/femoral chondrocytes showed more robust proliferation than phalangeal/metacarpal chondrocytes, in part due to differences in the Igf signaling pathway.

In addition to Igf signaling, our RNA-Seq analysis in the hypertrophic zone identified a number of genes implicated in bone morphogenetic protein (Bmp) signaling, another paracrine regulatory system that is important in the growth plate [18] (Fig 4C and S7 Fig, bottom panel). We confirmed with qPCR that tibia expressed higher levels of *Bmp6* (a Bmp ligand) and lower levels of *Bmp3* and *Bmper* (both Bmp functional antagonists) in the hypertrophic zone (Fig 4E). Because numerous previous studies have implicated Bmp signaling in promoting chondrocyte hypertrophy [19,20], we hypothesized that these differences in Bmp agonist and antagonist levels may contribute to a difference in BMP signaling and therefore the observed difference in chondrocyte hypertrophy between these bones. Consistent with this hypothesis, western blot showed that chondrocytes isolated from tibial and femoral growth plate have higher levels of phosphorylated Smad1/5/9 compared with chondrocytes from metacarpal and phalangeal growth plate, suggesting the presence of more active BMP signaling. To test this hypothesis further, we cultured whole neonatal mouse tibia and metatarsal bones in the presence or absence of exogenous Bmp2 (Fig 4J). We found that administration of Bmp2 significantly increased hypertrophic cell height in metatarsals (129% of controls, *p* < 0.001) to match that of untreated tibias. In contrast, Bmp2 did not significantly increase hypertrophic cell height in tibias (105%, *p* = 0.207). We next treated the bones with Noggin, a Bmp functional antagonist, and found that it significantly reduced hypertrophy in tibias (*p* < 0.001) but not in metacarpals (*p* = 0.161) (Fig 4K). Collectively, these findings suggest that the difference in endogenous Bmp signaling contributes to the difference in chondrocyte hypertrophy between different long bones.

Our expression data also implicated a third paracrine regulatory system that is important in the growth plate, Wnt signaling [21] (S7 Fig, middle panel). RNA-Seq, confirmed by qPCR, showed that the tibia expressed higher levels of Wnt ligands (*Wnt4*, *Wnt5a*, *Wnt5b*) and lower levels of functional Wnt antagonists (*Wif1* and *Dkk3*) compared with the phalanx (Fig 4F). Wnt5a expression also decreased with age, while Wif1 expression increased with age, and both genes showed more advanced changes in the phalanges compared with the tibias (Fig 4F). These expression data suggest lower Wnt signaling levels in the shorter bone, but the functional role in the differential growth between longer and shorter bones remains to be determined.

We also considered the possibility that growth plate senescence (loss of function and involution with age) and differences among bones might involve cellular senescence (an irreversible cell cycle arrest mechanism) [22]. However, RNA-Seq did not show consistent gene expression changes with age or among bones typical of cellular senescence (S8 Fig, left panel). Furthermore, staining for senescence-associated β-galactosidase did not identify any positive cells in the growth plate (S8 Fig, right panel).

## Discussion

To explore the mechanisms that underlie the dramatic differences in lengths of different bones, we compared growth plates in shorter bones (metacarpals and proximal phalanges) versus growth plates of longer bones (distal femurs and proximal tibias) in 2 species, the mouse and the rat, at the cellular and molecular levels. Both of the critical determinants of longitudinal bone growth rate—the chondrocyte proliferation rate per cell column and the height of the terminal hypertrophic chondrocyte—were lower in growth plates of smaller bones compared with longer bones. Some prior studies have attributed the differences in growth of different bones primarily to hypertrophic chondrocyte size [11,12], but our findings are consistent with older studies demonstrating an additional important contribution from differences in the proliferation rate [23–25].

Our current study shows that these differences in proliferation and hypertrophy between bones are attributable, in part, to differences in growth plate senescence, the normal developmental program that involves a progressive decline in growth plate function and a progressive involution of the growth plate cartilage. In the smaller bones, multiple structural and functional makers of senescence were more advanced compared with longer bones. LCM combined with RNA-Seq demonstrated that an extensive developmental genetic program, which underlies growth plate senescence, was more advanced in the shorter bones, thus contributing to the earlier senescence.

Previous studies have implicated multiple signaling pathways in the regulation of chondrocyte proliferation and hypertrophy. For example, chondrocyte proliferation is promoted by Indian hedgehog [26], IGF signaling [14], and Wnt signaling (through Wnt5a) [21] and is inhibited by fibroblast growth factor (FGF) signaling (through signal transducer and activator of transcription 1 [Stat1]) [27], while chondrocyte hypertrophy is stimulated by BMP signaling [19] and IGF signaling [28] and is suppressed by parathyroid hormone-related peptide (PTHrP) [29] and FGF signaling (through mitogen-activated protein kinase [MAPK]) [30]. In the current study, we showed expression and functional evidence that the differential growth between shorter and longer bones was attributable in part to differential modulation by IGF signaling and BMP signaling. Part of these differences are age independent, but they appear to reflect more advanced growth plate senescence in the shorter bones. It is likely that multiple other pathways also contribute to the differential growth rates of different bones.

In this paper, we use the terms “aging” and “senescence” to describe the loss of function and involution that occur in the growth plate with increasing age [31]. Unlike age-related changes in other tissues that are typically described by these terms, these changes in the growth plate occur in the early portion of the lifespan, presumably provide survival advantages to the organism, and thus represent a developmental rather than a degradative process. The term “senescence” is also often used in the biological literature to describe a specific cellular differentiation pathway associated with increased expression of senescence-associated β-galactosidase and p16 [22]. Our findings suggest that the physiological senescence occurring in the growth plate does not involve this cellular process of senescence.

**Fig 5:**
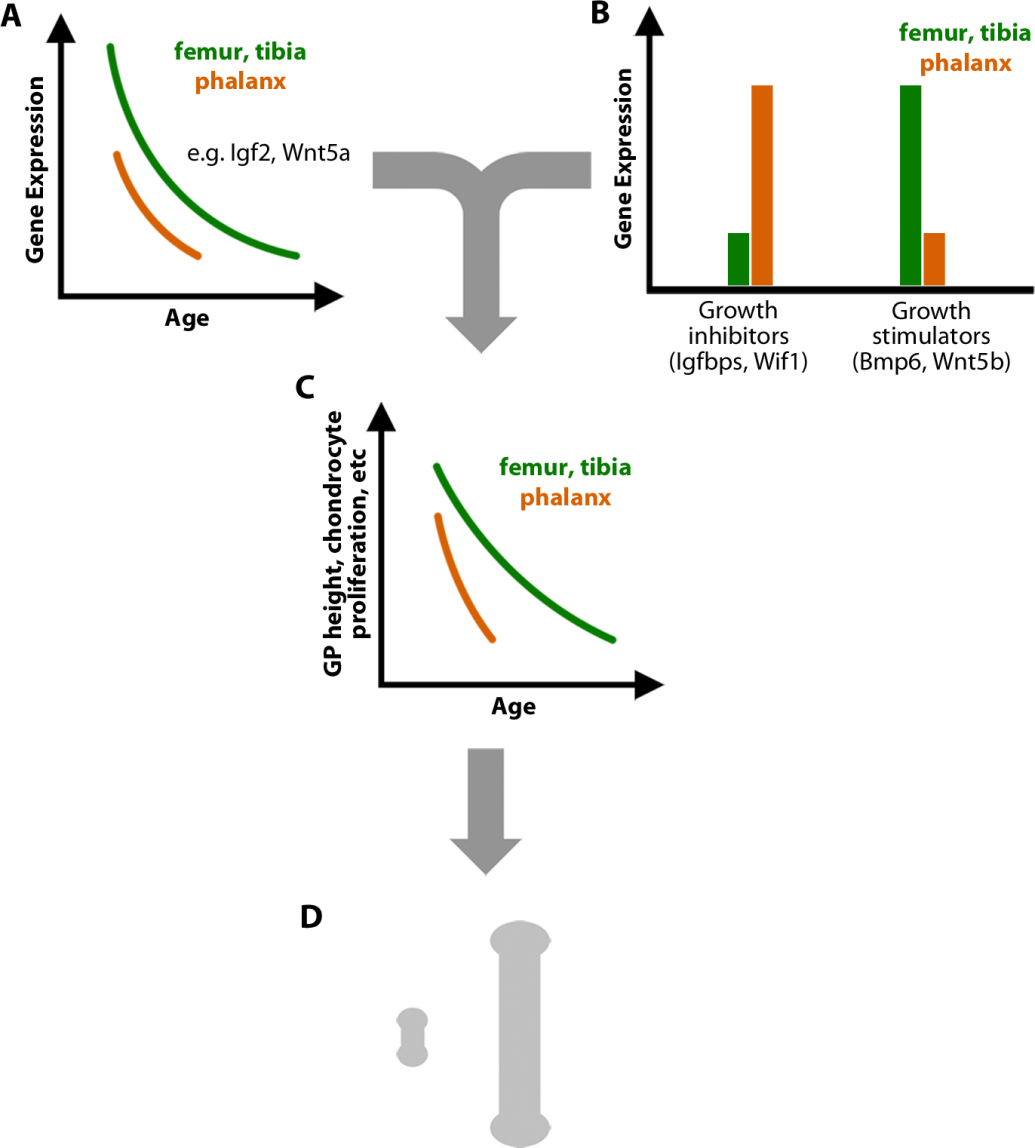
Differences in bone length arise in part from modulation of the developmental program of growth plate senescence. (A) Senescent changes in gene expression in the growth plate, including genes encoding many paracrine signaling molecules, are more advanced in the shorter bones. (B) Age-independent differences in gene expression within key regulatory pathways also contribute to differences in growth rate. (C) Consequently, the developmental program of growth plate senescence, including structural involution and declines in proliferation and hypertrophic cell size, is more advanced in shorter bones. (D) Differences in proliferation rate and hypertrophic cell size result in disparities in the rate of bone elongation and thus cumulative bone length. Bmp, bone morphogenetic protein; GP, growth plate; IGF, insulin-like growth factor; Igfbp, IGF-binding protein; Wif, Wnt inhibitory factor; Wnt, Wingless and Int-1.

Taken together, our findings support the following model to explain the dramatic disparities in length and growth rate of different long bones (Fig 5). In all growth plates, a development genetic program occurs that involves changes in expression of many genes important for the regulation of chondrocyte growth, including key transcription factors and important paracrine signaling molecules such as Igf, Bmp, and Wnt ligands. These changes in gene expression occur earlier in the shorter bones, such that the program is more advanced at any given age (Fig 5A). In addition to these senescent changes, there are age-independent differences in gene expression between growth plates from different bones (Fig 5B). In some cases, these differences, such as the higher levels of Igfbps in the phalanges, render the shorter bones more responsive to the early senescent changes (like declining paracrine signaling by Igf2). The findings suggest that the interaction between these age-dependent and age-independent differences in expression contribute to accelerated growth plate senescence, lower growth rates, and earlier growth cessation in the phalanges and metacarpals (Fig 5C), which underlie the dramatic reduction in final length of these bones compared with femurs and tibias (Fig 5D). The relative contribution of age-dependent and age-independent mechanisms remains to be determined. In summary, our findings reveal that the size disparities between different bones arise in part from modulation of the developmental program of growth plate senescence.

## Methods

### Ethics statement

All animal procedures were approved by the National Institute of Child Health and Human Development Animal Care and Use Committee. The approved animal protocol number is 18–031. Mice or rats up to 10 days of age were euthanized by decapitation according to the NIH Animal Research Advisory Committee (ARAC) guidelines. Older mice or rats were euthanized by carbon dioxide inhalation in a chamber. Our current study did not involve any human participants or human tissues.

### Animals

C57BL/6 mice were obtained from Charles River Laboratory, and Sprague Dawley rats were obtained from Envigo (Frederick, MD). A combination of male and female mice were used for embryonic and newborn time points, and only male mice and rats were used in all other time points and experiments. Comparisons were made between the growth plates of the distal femur, proximal tibia, distal metacarpal, and proximal end of the proximal phalanx of the forelimb.

### Growth plate dissection

After animals were euthanized, femoral, tibial, metacarpal, and phalangeal epiphyses were excised. For LCM, cartilage was embedded in optimum cutting temperature (OCT) compound (Electron Microscopy Sciences, Hatfield, PA), frozen on dry ice, and stored at −80°C. LCM of growth plate cartilage was performed as previously described [32]. For histology, cartilage was fixed overnight in formalin at 4 °C and decalcified in 10% (w/v) EDTA (pH 7.4). Samples were then embedded in paraffin for sectioning. Masson Trichrome–stained histological sections were prepared by Histoserv (Germantown, MD).

### RNA extraction and purification

RNA was extracted using an RNeasy Micro Kit (QIAGEN, Valencia, CA). All RNA samples had a 260/280 nm ratio between 1.8 and 2.1. RNA integrity was determined using an Agilent 2100 Bioanalyzer (Agilent Technologies, Santa Clara, CA), and only high-quality RNA (RIN >7) was used for RNA-Seq or real-time qPCR.

### qPCR

Real-time PCR was used to assess mRNA levels in different zones of different growth plates at various ages. Total RNA (50–100 ng) was reverse-transcribed using SuperScript IV Reverse Transcriptase (Invitrogen). Real-time qPCR was performed as previously described [33] using commercially available FAM- or VIC-labeled Taqman assays (Applied Biosystems, Foster City, CA). Reactions were performed in triplicate on cDNA derived from each animal using the ABI QuantStudio 6 Flex System instrument (Applied Biosystems). The relative quantity of each mRNA was calculated using the formula Relative Expression = 2^−ΔCt^ × 10^6^, where Ct represents the threshold cycle and ΔCt = (Ct of gene of interest) − (Ct of 18S rRNA). Values were multiplied by 10^6^ for convenience of comparison. A list of all taqman probes used was given in S11 Table.

### RNA-Seq

For each animal, total RNA from proliferative zone or hypertrophic zone (*n* = 5 each) was amplified using Ovation RNA-Seq System V2 (NuGEN, San Carlos, CA). RNA-Seq libraries were then constructed using Nextera XT DNA Library Preparation Kit (Illumina, San Diego, CA) and sequenced on HiSeq 3000 System (Illumina). Approximately 40 million 50 bp paired-end reads were generated from each sample. Alignment of RNA-Seq data was performed with RNA-STAR (version 2.4.2) [34] against the mouse mm10 genome build and rat rn6 genome build. Gene-based read quantitation was performed using SubRead featureCounts (version 1.4.6) [35]. Read counts were normalized by Relative Log Expression (RLE) method [36] and evaluated for differential expression using the Bioconductor package DESeq2 [37], comparing defined sample sets as biological replicates and drawing pairwise comparisons.

### Growth plate quantitative histology

Histological evaluations were performed on Masson Trichrome–stained epiphyseal sections that were visualized using a ScanScope CS digital scanner (Aperio Technologies) under bright field microscopy. All histological measurements were performed in the central two-thirds of the growth plate sections as previously described [38]. Briefly, eights were measured parallel to the chondrocyte columns. Hypertrophic chondrocytes were operationally defined by a height ≥10 μm. The terminal hypertrophic chondrocyte was defined as the cell in the last lacuna that was not invaded by metaphyseal blood vessels. Resting zone was not well-defined before postnatal 1 week and therefore was not measured. At age 1 week, the resting zone was defined as starting from the first non-hypertrophic cells below the location of the future secondary ossification center and continuing down to the slightly flat doublet chondrocytes before formation of long columns of proliferative chondrocytes. From 2 weeks onward, the beginning of the resting zone was defined as the lower margin of the secondary ossification center. Numbers of cells in the resting zone were counted per 500 μm growth plate width. For each growth plate section, we performed at least 3 measurements of proliferative zone height, hypertrophic zone height, and terminal hypertrophic cell height. For terminal hypertrophic cell height, the height of the lacunae—which reflects the actual hypertrophic chondrocyte height before cells condense during tissue fixation and processing—was measured. We also counted the number of proliferative and hypertrophic cells in at least 3 intact columns from each growth plate section. For each animal, averages were taken from 8 growth plate sections.

### BrdU staining and analysis

The proliferation rate was determined by BrdU staining. BrdU was injected (0.1 mg/g body mass intraperitoneally; Sigma-Aldrich, St. Louis, MO) 2 hours before mice were killed, and the growth plates were dissected, fixed, and decalcified. Samples were embedded in paraffin, and 10 μm sections were mounted on Superfrost Plus slides. BrdU labeling was detected by immunohistochemistry using the BrdU In-Situ Detection Kit (BD Biosciences, San Jose, CA) and counterstained with methyl green. BrdU-positive cells were counted in the center two-thirds of the growth plate proliferative zone. BrdU-positive cells per column was calculated by dividing the total number of BrdU-positive cells by the number of proliferative columns in the counted area. BrdU labeling index was calculated by dividing the total number of BrdU-positive cells by the total number of cells in the proliferative zone in the counted area. Position-specific BrdU labeling index was calculated at each position of the proliferative column, with the first columnar cell underneath the resting zone defined as position 1, by dividing the number of BrdU-positive cells at each position by the total number of cells at that same position in the counted area.

### Assessment of bone growth rate by calcein injection

To assess the rate of physical bone growth, we injected calcein intraperitoneally in mice at various ages [39]. Mice were euthanized 48 hours after injection, and proximal tibias, distal femurs, metacarpals, and proximal phalanges were dissected and fixed in formalin. The undecalcified bones were embedded in paraffin or glycol methacrylate for sectioning. Bone sections were rehydrated and counterstained with DAPI (4',6-diamidino-2-phenylindole) and mounted with Fluoromount G (Thermo Fisher). Calcein incorporates into bone matrix and fluoresces green [40]. However, because calcein has a short half-life in circulation, the bone formed below the growth plate in the 48 hours after calcein injection will not be labeled. The amount of longitudinal bone growth was therefore measured as the distance between the green fluorescence in the metaphysis and the chondro-osseous junction, observed and analyzed using a Keyence BZ-X700 fluorescence microscope (Keyence Corp, Osaka, Japan) at 5X magnification. For example, the bone growth rate (μm/d) for 7-day-old mice was measured by injecting calcein at 6 days of age and euthanizing at 8 days of age and dividing the distance between green fluorescence and chondro-osseous junction by 2.

### Staining of senescence-associated beta-galactosidase

To assess cellular senescence in the growth plate, freshly dissected growth plates were embedded in OCT and snap-frozen on dry ice and maintained at −20 °C. Frozen sections of growth plate were immediately prepared, fixed for 1 minute in 5% formalin, and incubated overnight at 37  °C in 1 mg/ml of X-gal (5-bromo-4-chloro-3-indolyl β-D-galactopyranoside; Invitrogen), 5 mM K_3_Fe(CN)_6_ (Sigma-Aldrich), 5 mM K_4_Fe(CN)_6_×3H_2_O (Sigma), and 40 mM citric acid/sodium phosphate (pH 6.0) as previously described [41]. The reaction was stopped by washing the slides in PBS (pH 7.4) and fixing in 10% formalin, followed by counterstaining with 0.1% nuclear fast red solution (Sigma), dehydrating in serial ethanol solutions (70%, 85%, and 100%, 5 minutes each), immersing in xylene, and mounting using Permount solution (Fisher Scientific).

### Chondrocyte isolation and culture

Growth plates from proximal phalanges, distal metacarpals, proximal tibias, and distal femurs were dissected from 1-week-old mice aseptically and digested in 0.3% collagenase type I (Sigma-Aldrich) in DMEM/F12 medium. Proximal phalanges were combined with distal metacarpals, while proximal tibias were combined with distal femurs. The released cells were resuspended and plated at a density of 1 × 10^5^ chondrocytes per well in 12-well plates, in DMEM/F12 medium (Invitrogen) supplemented with 10% fetal bovine serum (FBS), 1% penicillin (100 U/ml)/streptomycin (100 μg/ml), and 50 μg/mL ascorbic acid in a humidified incubator at 37 °C, 5% CO_2_.

### Western blot

Protein was isolated from chondrocytes from mouse tibial and femoral growth plate or metacarpal and phalangeal growth plate using RIPA buffer supplemented with proteinase inhibitor cocktail (Sigma-Aldrich) and PhosSTOP (Sigma-Aldrich). Cell lysates were incubated on ice for 30 minutes, followed by a 10-minute centrifugation at 21,000 g to remove cell debris. Western blotting was performed as previously described [42] using either phospho-SMAD1/5/9 (Cell Signaling, number 13820) or total SMAD1/5/9 (Abcam, ab80255).

### Proliferation assessment by ^3^H-thymidine uptake

To assess cell proliferation, chondrocytes were incubated with 1 ml of fresh culture medium containing 1 μCi of ^3^H-thymidine (63 Ci/mmol; MP Biomedicals, Santa Ana, CA) for 16 hours and then vigorously washed 3 times with PBS. Chondrocytes were detached by collagenase digestion (0.3%, 30 minutes), and radioactivity was measured by liquid scintillation counting.

### Transfection of chondrocytes with siRNA

Transfection was performed as previously described [38]. Briefly, monolayer chondrocytes were treated with hyaluronidase (5 U/mL; Sigma-Aldrich) for 6 hours. Prior to transfection, cells were washed once in PBS and changed to DMEM/F12 medium without antibiotics. Negative-control siRNA or siRNA against Igf2 (SMARTpool siGENOME Igf2 siRNA; Dharmacon, Lafayette, CO) was transfected (40 pmol per reaction) into chondrocytes using Lipofectamine 2000 (Life Technologies) following the manufacturer’s standard protocol.

### Combined siRNA and Igf1 treatment of chondrocytes

On day 1, primary chondrocytes were isolated and cultured at 1 × 10^5^ cells per well in 12-well plates. On day 2, cells were treated with hyaluronidase and transfected 6 hours later with control siRNA or siRNA against Igf2. On day 3, culture medium was changed to DMEM/F12 without FBS. On day 4, chondrocyte culturing continued in serum-deprived medium and were treated with Igf1 (100 ng/mL). ^3^H-thymidine was added to the culture medium 16 hours before proliferation was assessed on day 5.

### Tibia and metatarsal organ culture

Mouse tibia and metatarsal culture was performed as previously described [38,43]. Briefly, whole tibias and the middle 3 metatarsals were aseptically dissected from newborn C57BL/6 mice. Bones were maintained in 500 μlαMEM medium supplemented with 0.2% bovine serum albumin, 0.1 mM β-glycerophosphate, 50 μg/ml ascorbic acid, 1% penicillin/streptomycin, and 0.1% Fungizone. Bone were cultured individually in 24-well plates in a humidified incubator at 37 °C, 5% CO_2_ for 3 days. Medium was refreshed on day 2 of culture. Recombinant mouse Bmp2 (100 ng/mL; R&D systems) or noggin (200 ng/mL; R&D systems) was added at the beginning of culture and refreshed on day 2. All bones were collected after 3 days of culture, fixed in formalin, and decalcified for histological sectioning.

### Statistics

Data are presented as mean ± SEM. Scatterplots, correlations, heatmaps, and hierarchical clustering were performed using JMP 13 software (SAS Institute, Cary, NC). Signaling pathway analysis was performed using Ingenuity Pathways Analysis Software (Ingenuity Systems, Redwood City, CA), and gene ontology analysis was performed using the Gene Ontology Consortium web interface [44,45]. ANOVA analyses were performed in SigmaPlot 11. One-way ANOVA was used when measuring the effect of a single factor, such as effect of Igf1 on proliferation. Two-way ANOVA was used when the effect of more than one factor was being assessed, such as when comparing the effect of age on gene expression between tibia and phalanx. *p*-Values were corrected for multiple comparisons, whenever applicable, using the Holm-Sidak method.

## Supporting information

S1 Fig Growth plates of mouse metacarpals and phalanges show earlier declines in chondrocyte proliferation compared with tibias and femurs. Mice received BrdU to label proliferating cells, and BrdU was visualized (brown color) by immunohistochemistry with methyl green counterstain. BrdU staining of metacarpal and phalanx was not performed at time points beyond 3 weeks old due to growth plate fusion. Scale bar, 50 μm. BrdU, 5-bromo-2-deoxyuridine.
(TIF)

S2 Fig Growth plate senescence is more advanced in shorter bones than in longer bones in rats. (A) Masson Trichrome–stained histological sections of proximal phalanges, metacarpals, proximal tibias, and distal femurs from Sprague-Dawley rats at various postnatal ages. Cartilage matrix stains light blue; bone matrix, dark blue. Epiphyseal fusion (disappearance of growth plate) occurs at approximately 12 weeks in phalanges and 16 weeks in metacarpals but has not yet occurred at 16 weeks in tibias or femurs. Scale bar, 100 μm. (B–I) Quantitative histological measurements of RZ height (panel B) and cell count (panel C); PZ height (panel D), cell count per column (panel E), and cell proliferation rate (panel F); HZ height (panel G), cell count per column (panel H), and terminal hypertrophic cell height (panel I), in each of the 4 growth plates at various ages. *N* = 6, mean ± SEM. Raw values are available in S1 Data. HZ, hypertrophic zone; PZ, proliferative zone; RZ, resting zone.
(TIF)

S3 Fig **BrdU-labeling indices (BrdU-positive cells/total cells) of proliferative zone of proximal tibias, distal femurs, distal metacarpals, and proximal forelimb phalanges in mice (left panel) and rats (right panel).** All raw values are available in S1 Data. BrdU, 5-bromo-2-deoxyuridine.
(TIF)

S4 Fig Position-specific BrdU-labeling indices of proliferative zone of proximal tibias, distal femurs, distal metacarpals, and proximal forelimb phalanges in mice at various ages. Cell position 1 denotes the proliferative zone chondrocyte closest to the resting zone. Black arrow indicates the average cell position where the proliferative zone ends. Raw values are available in S1 Data. BrdU, 5-bromo-2-deoxyuridine.
(TIF)

S5 Fig Validation of LCM with zonal markers of the postnatal growth plate. RNA-Seq was performed on laser capture micodissected PZ or HZ of 1-week proximal tibia (top panel), 1-week proximal phalanges (middle panel), and 4-week proximal (bottom panel). Log_2_ (normalized raw counts) in the PZ and HZ of genes previously identified [7] to be expressed specifically in the PZ (Gdf10, Prelp, Bmp7) or HZ (Col10a1, Bmp2, Mmp13) were used to confirm the accuracy of our dissection. Raw values are available in S1 Data. HZ, hypertrophic zone; LCM, laser capture microdissection; PZ, proliferative zone; RNA-Seq, RNA sequencing.
(TIF)

S6 Fig Schematic diagram depicting how differences in the timing of growth plate senescence between different bones could cause a correlation between age-related changes in gene expression and bone-related differences in gene expression. We hypothesized that growth plate senescence and the underlying changes in gene expression are more advanced in the shorter bones, thus explaining their slower growth rate and diminished length. This hypothesis predicts that the age-dependent changes in gene expression would be more advanced in the phalanges than in the tibias. Consequently, for genes that showed decreasing expression with age in the tibia, the expression would be lower in 1-week phalanges than in 1-week tibias (panel A). Conversely, for genes that showed increasing expression with age in the tibia, the expression would be greater in 1-week phalanges than in 1-week tibias (panel B). Thus, one would expect a positive correlation between changes in gene expression with age in the tibias (fold change, 1 week versus 4 weeks) and differences in gene expression between the bones (fold difference, tibias versus phalanges) at 1 week (panel C). The data testing this relationship are shown in Fig 3A and 3B.
(TIF)

S7 Fig Heatmaps showing expression of principal genes involved in IGF, WNT, and BMP signaling, analyzed by RNA-Seq, in proliferative or hypertrophic zones of 1- and 4-week tibia and 1-week phalanx. Genes were arranged by functional categories rather than by hierarchical clustering. Ligands, green; receptors, black; functional antagonists, red. Scale bar represents log_2_ (fold differences). Raw values used to generate the heatmaps are available in S1 Data. BMP, bone morphogenetic protein; IGF, insulin-like growth factor; RNA-Seq, RNA sequencing; WNT, Wingless and Int-1.
(TIF)

S8 Fig Physiological growth plate senescence (loss of function and involution with age) does not appear to involve cellular senescence (an irreversible cell cycle arrest mechanism). Left panels: markers of cellular senescence (genes that are known to show increased expression in senescent cells) were analyzed by RNA-Seq in proliferative and hypertrophic zones of 1- and 4-week tibia and 1-week phalanx. Scale bar represents log_2_ (fold differences). Raw values used to generate the heatmaps are available in S1 Data. Right panels: senescence-associated beta-galactosidase, which is a widely used marker for cellular senescence, was examined by X-gal staining in freshly frozen sections of 1- and 4-week tibias and 1-week metacarpal/phalanges. Scale bar, 100 μm. RNA-Seq, RNA sequencing.
(TIF)

S1 Table Relative difference in terminal hypertrophic cell height, chondrocyte proliferation in the proliferative zone, and physical bone growth rate between 4 different mouse growth plates at newborn, postnatal 1, 2, and 3 weeks. Tibia was used as the denominator.
(XLSX)

S2 Table Relative difference in terminal hypertrophic cell height and chondrocyte proliferation in the proliferative zone between 4 different rat growth plates at postnatal 1, 2, 4, and 8 weeks. Tibia was used as the denominator.
(XLSX)

S3 Table List of 200 genes significantly up- or down-regulated with age in tibia proliferative zone. Selection criteria: >2-fold, FDR < 0.05, in both species. Log_2_ (fold changes) were used; *p*-Values were corrected for multiple comparisons. FDR, false discovery rate.
(XLSX)

S4 Table List of 129 genes significantly up- or down-regulated with age in tibia hypertrophic zone. Selection criteria: >2-fold, FDR < 0.05, in both species. Log_2_ (fold changes) were used; *p*-Values were corrected for multiple comparisons. FDR, false discovery rate.
(XLSX)

S5 Table List of 161 genes differentially expressed between tibia and phalanx proliferative zone at 1 week of age. Selection criteria: >2-fold, FDR < 0.05, in both species. Log_2_ (fold changes) were used; *p*-Values were corrected for multiple comparisons.
(XLSX)

S6 Table List of 403 genes differentially expressed between tibia and phalanx hypertrophic zone at 1 week of age. Selection criteria: >2-fold, FDR < 0.05, in both species. Log_2_ (fold changes) were used; *p*-Values were corrected for multiple comparisons.
(XLSX)

S7 Table Gene ontology terms significantly enriched in the list of 161 PZ-bone–specific genes (S3 Table). Selection criteria: >3-fold enrichment, *p* < 0.05, corrected for multiple comparisons. PZ, proliferative zone.
(XLSX)

S8 Table Gene ontology terms significantly enriched in the list of 403 HZ-bone–specific genes (S4 Table). Selection criteria: >3-fold enrichment, *p* < 0.05, corrected for multiple comparisons. HZ, hypertrophic zone.
(XLSX)

S9 Table Canonical signaling pathway significantly implicated in the list of 161 PZ-bone–specific genes (S3 Table). Selection criteria, *p* < 0.05 by Fisher’s exact test. PZ, proliferative zone.
(XLSX)

S10 Table Canonical signaling pathway significantly implicated in the list of 403 HZ-bone–specific genes (S4 Table). Selection criteria, *p* < 0.05 by Fisher’s exact test. HZ, hypertrophic zone.
(XLSX)

S11 Table List of taqman probes used for real-time qPCR in our study. qPCR, quantitative PCR.
(XLSX)

S1 Data Contains all the raw quantitative data values used to generate Figs 1C and 1D, 2B–2G, 3A, 3B, 3I and 3J, 4D–4H and 4K, and S2B–S2I, S3, S4, S5, S7 and S8 Figs. (XLSX)

## Abbreviations

BMP: bone morphogenetic protein
BrdU: 5-bromo-2-deoxyuridine
FBS: fetal bovine serum
FDR: false discovery rate
FGF: fibroblast growth factor
IGF: insulin-like growth factor
Igfbp: IGF-binding protein
LCM: laser capture microdissection
MAPK: mitogen-activated protein kinase
OCT: optimum cutting temperature
PTHrP: parathyroid hormone-related peptide
qPCR: quantitative PCR
RLE: Relative Log Expression
RNA-Seq: RNA sequencing
siRNA: small interfering RNA
Stat1: signal transducer and activator of transcription 1
Wif: Wnt inhibitory factor
Wnt: Wingless and Int-1
