# Supplementary figures and images for: Differential aging of growth plate cartilage underlies differences in bone length and thus helps determine skeletal proportions
Source: PLoS Biol. 2018 Jul 23;16(7):e2005263. doi: 10.1371/journal.pbio.2005263 (PMC6056026; doi:10.1371/journal.pbio.2005263)

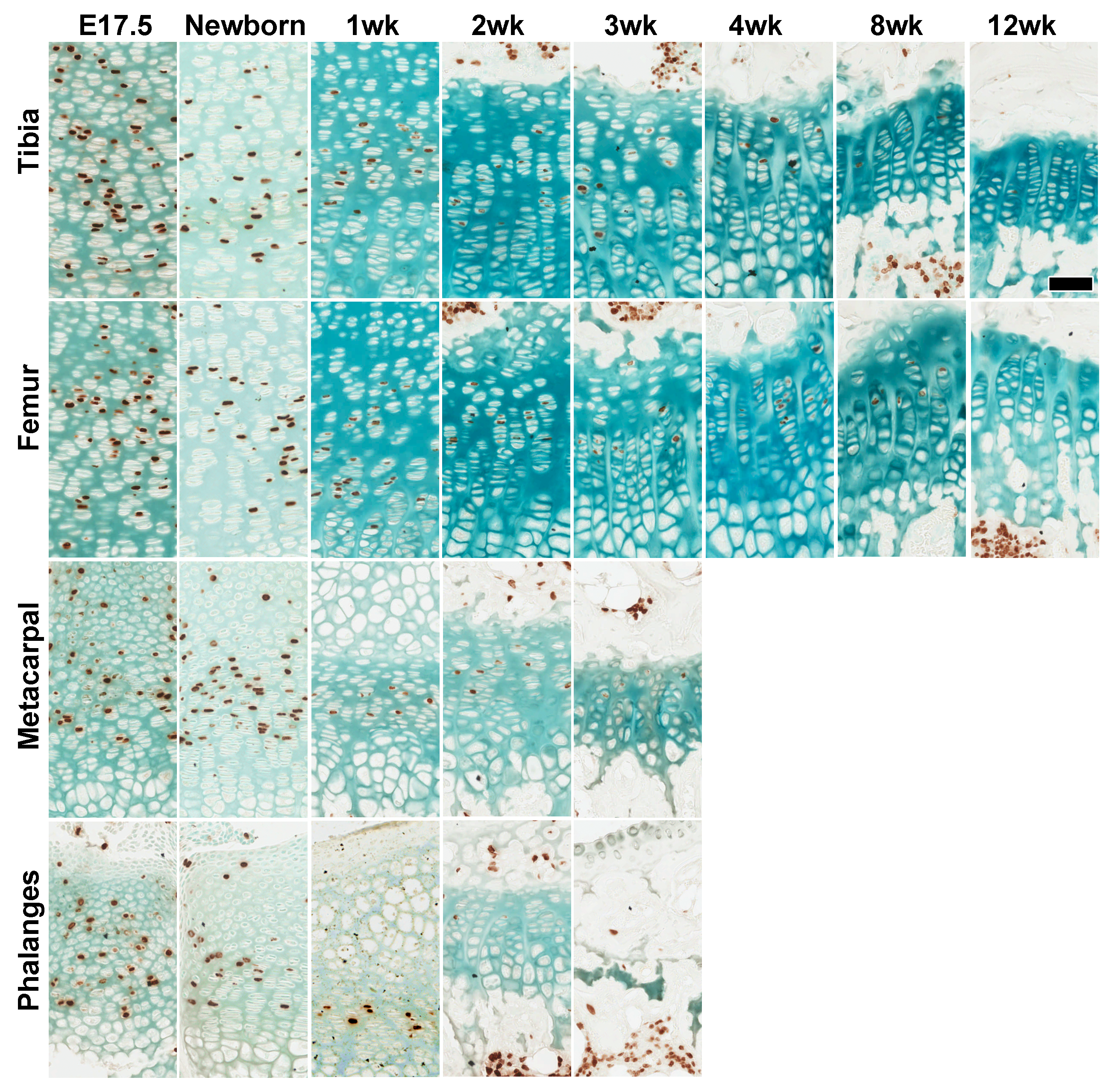

Supplement: S1 Fig — Mice received BrdU to label proliferating cells, and BrdU was visualized (brown color) by immunohistochemistry with methyl green counterstain. BrdU staining of metacarpal and phalanx was not performed at time points beyond 3 weeks old due to growth plate fusion. Scale bar, 50 μm. BrdU, 5-bromo-2-deoxyuridine. (TIF) [file pbio.2005263.s001.tif]

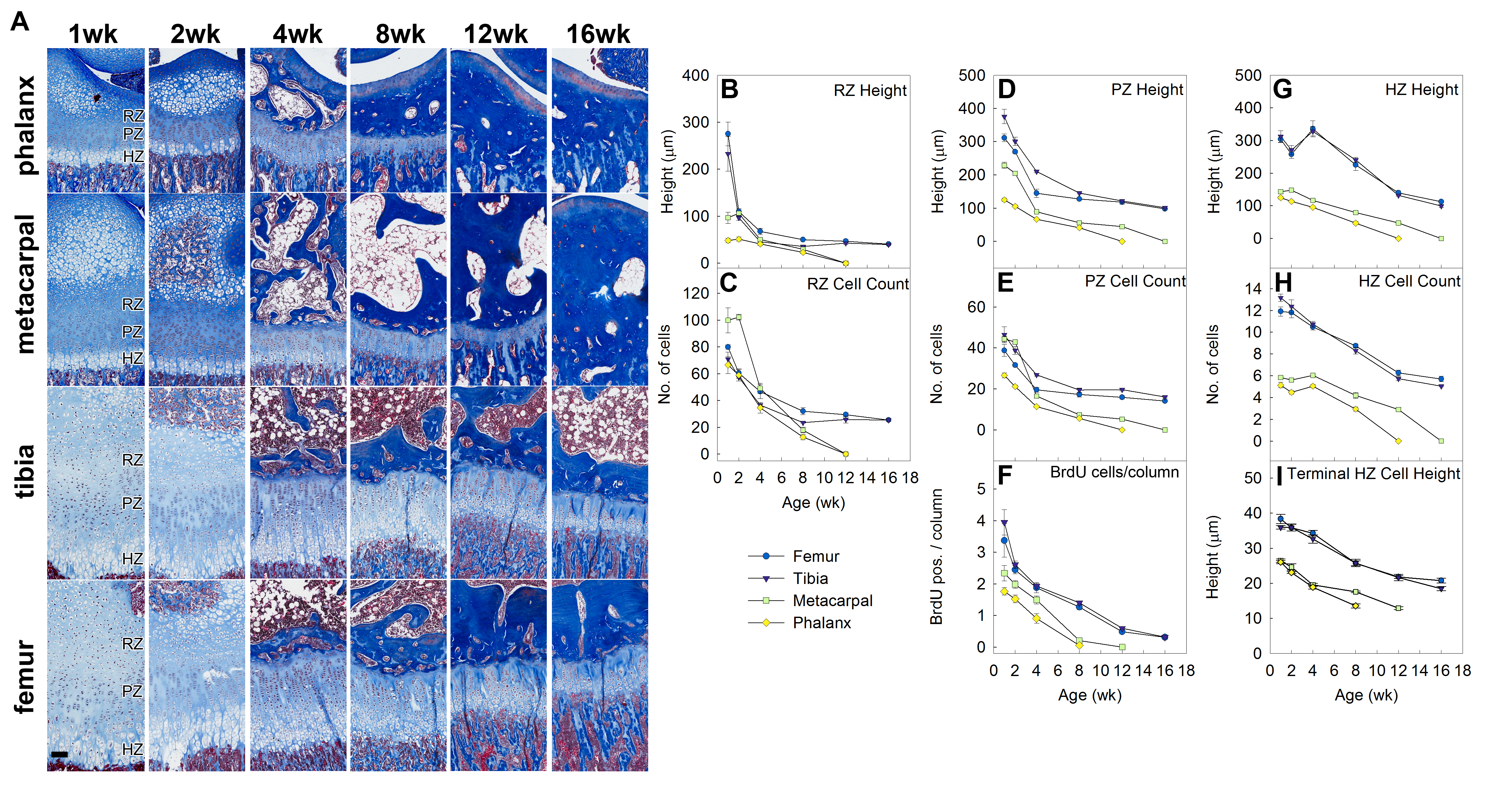

Supplement: S2 Fig — (A) Masson Trichrome–stained histological sections of proximal phalanges, metacarpals, proximal tibias, and distal femurs from Sprague-Dawley rats at various postnatal ages. Cartilage matrix stains light blue; bone matrix, dark blue. Epiphyseal fusion (disappearance of growth plate) occurs at approximately 12 weeks in phalanges and 16 weeks in metacarpals but has not yet occurred at 16 weeks in tibias or femurs. Scale bar, 100 μm. (B–I) Quantitative histological measurements of RZ height (panel B) and cell count (panel C); PZ height (panel D), cell count per column (panel E), and cell proliferation rate (panel F); HZ height (panel G), cell count per column (panel H), and terminal hypertrophic cell height (panel I), in each of the 4 growth plates at various ages. N = 6, mean ± SEM. Raw values are available in S1 Data. HZ, hypertrophic zone; PZ, proliferative zone; RZ, resting zone. (TIF) [file pbio.2005263.s002.tif]

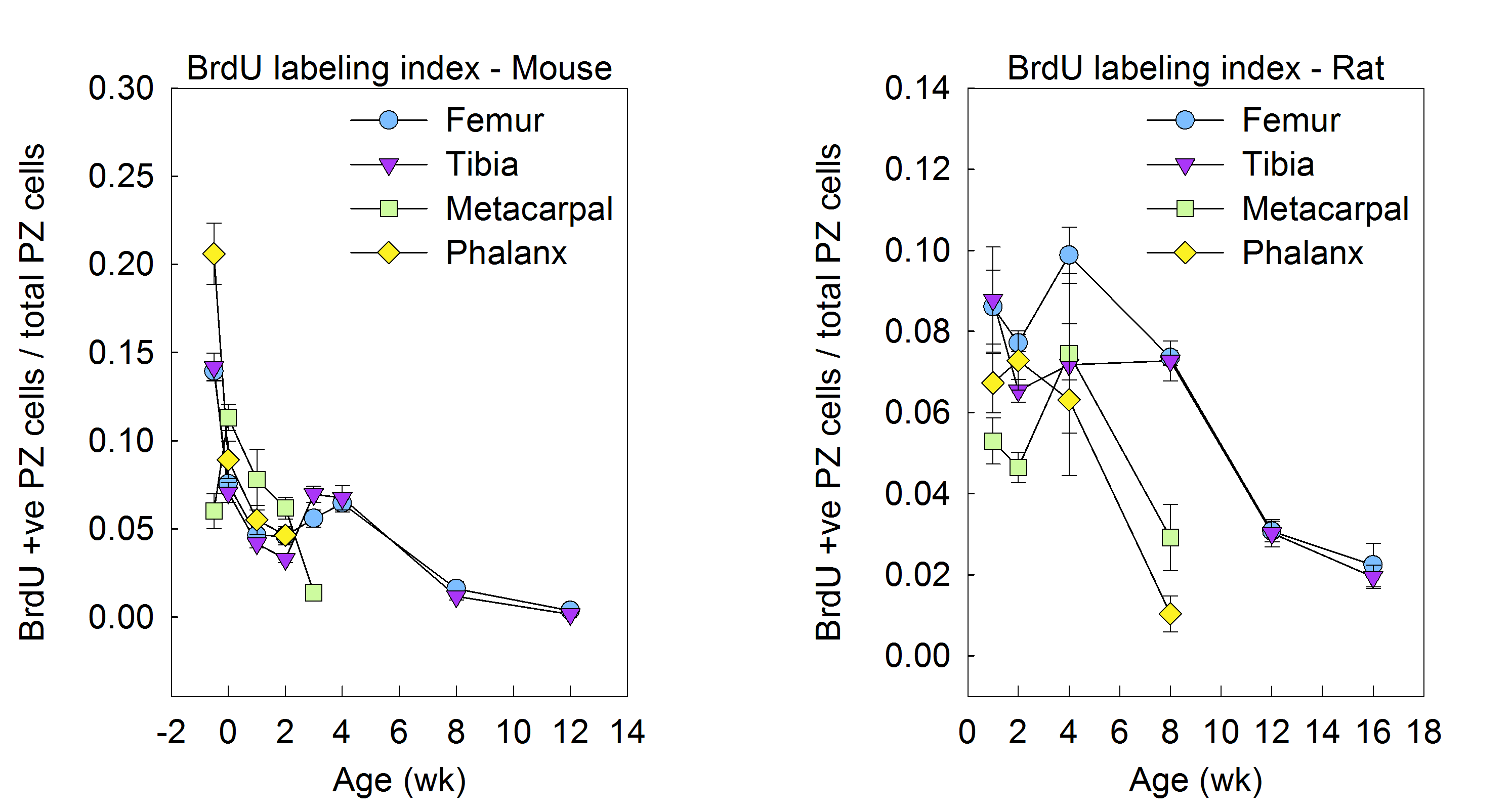

Supplement: S3 Fig — BrdU-labeling indices (BrdU-positive cells/total cells) of proliferative zone of proximal tibias, distal femurs, distal metacarpals, and proximal forelimb phalanges in mice (left panel) and rats (right panel). All raw values are available in S1 Data. BrdU, 5-bromo-2-deoxyuridine. (TIF) [file pbio.2005263.s003.TIF]

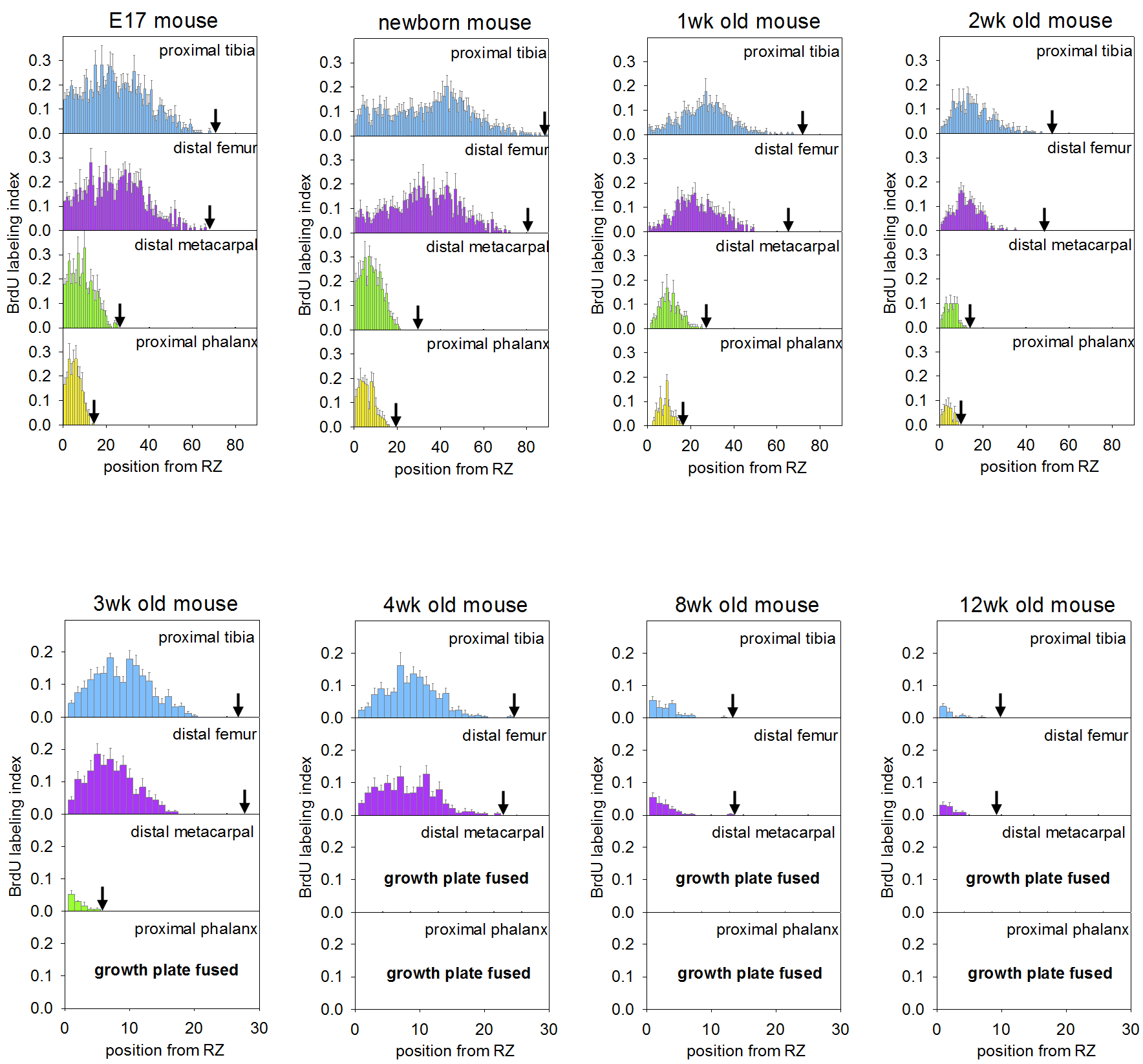

Supplement: S4 Fig — Cell position 1 denotes the proliferative zone chondrocyte closest to the resting zone. Black arrow indicates the average cell position where the proliferative zone ends. Raw values are available in S1 Data. BrdU, 5-bromo-2-deoxyuridine. (TIF) [file pbio.2005263.s004.TIF]

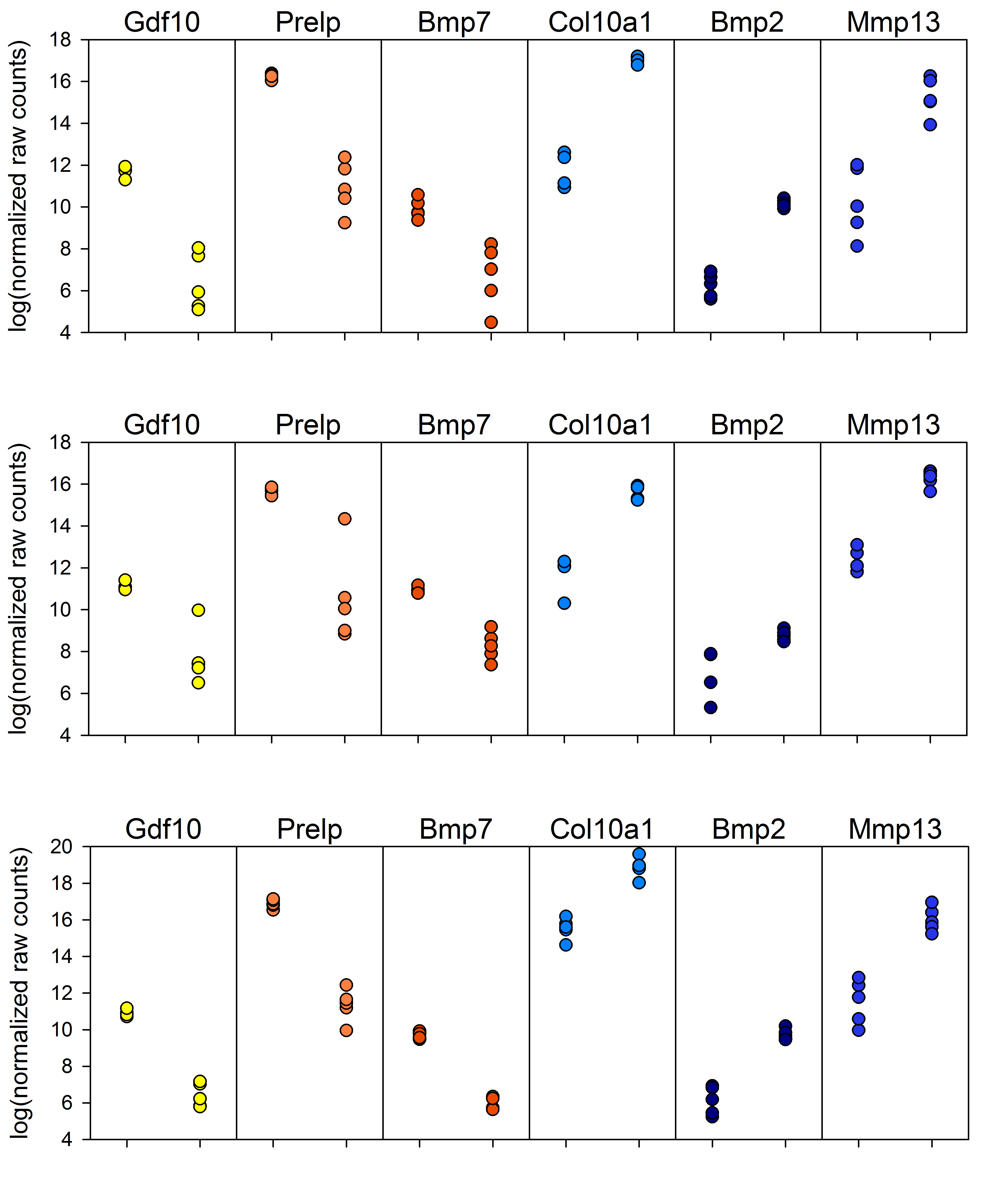

Supplement: S5 Fig — RNA-Seq was performed on laser capture micodissected PZ or HZ of 1-week proximal tibia (top panel), 1-week proximal phalanges (middle panel), and 4-week proximal (bottom panel). Log2 (normalized raw counts) in the PZ and HZ of genes previously identified [7] to be expressed specifically in the PZ (Gdf10, Prelp, Bmp7) or HZ (Col10a1, Bmp2, Mmp13) were used to confirm the accuracy of our dissection. Raw values are available in S1 Data. HZ, hypertrophic zone; LCM, laser capture microdissection; PZ, proliferative zone; RNA-Seq, RNA sequencing. (TIF) [file pbio.2005263.s005.TIF]

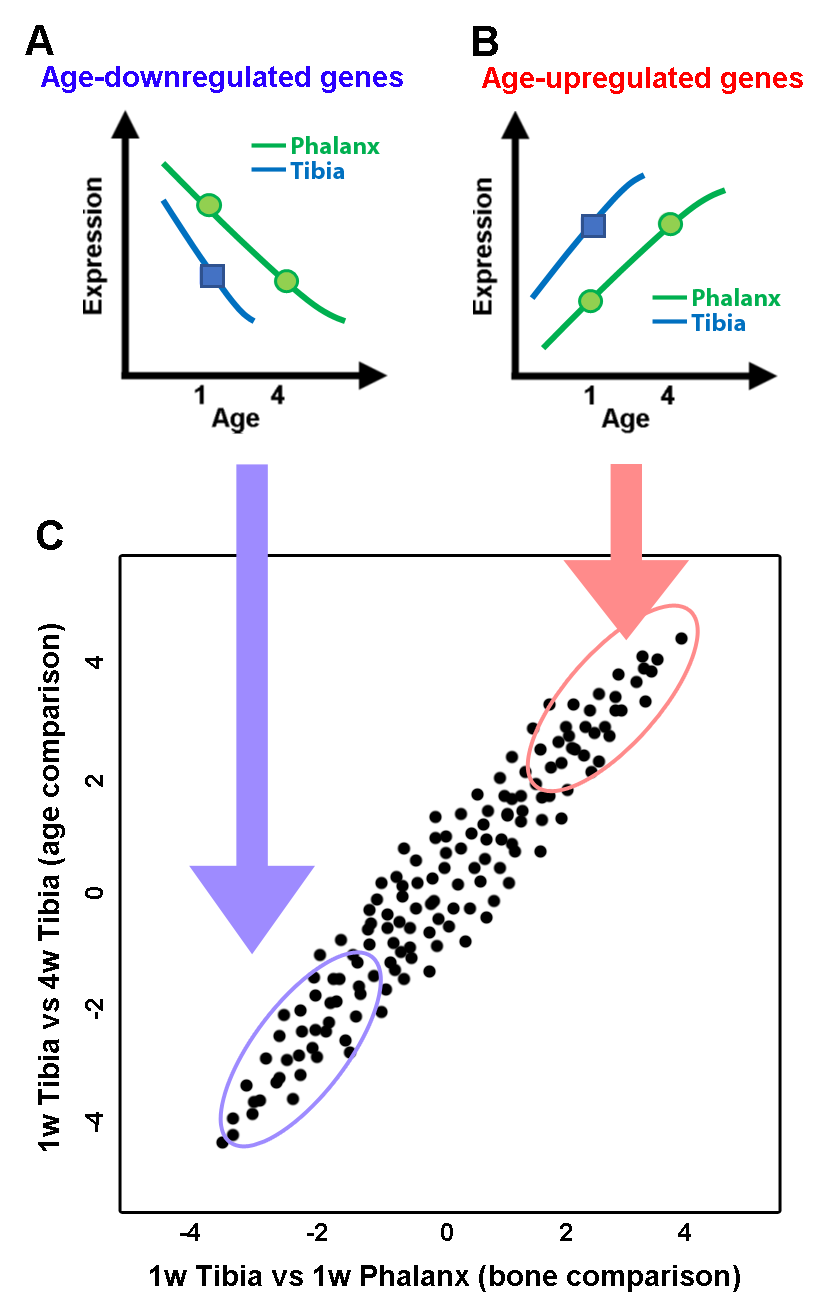

Supplement: S6 Fig — We hypothesized that growth plate senescence and the underlying changes in gene expression are more advanced in the shorter bones, thus explaining their slower growth rate and diminished length. This hypothesis predicts that the age-dependent changes in gene expression would be more advanced in the phalanges than in the tibias. Consequently, for genes that showed decreasing expression with age in the tibia, the expression would be lower in 1-week phalanges than in 1-week tibias (panel A). Conversely, for genes that showed increasing expression with age in the tibia, the expression would be greater in 1-week phalanges than in 1-week tibias (panel B). Thus, one would expect a positive correlation between changes in gene expression with age in the tibias (fold change, 1 week versus 4 weeks) and differences in gene expression between the bones (fold difference, tibias versus phalanges) at 1 week (panel C). The data testing this relationship are shown in Fig 3A and 3B. (TIF) [file pbio.2005263.s006.tif]

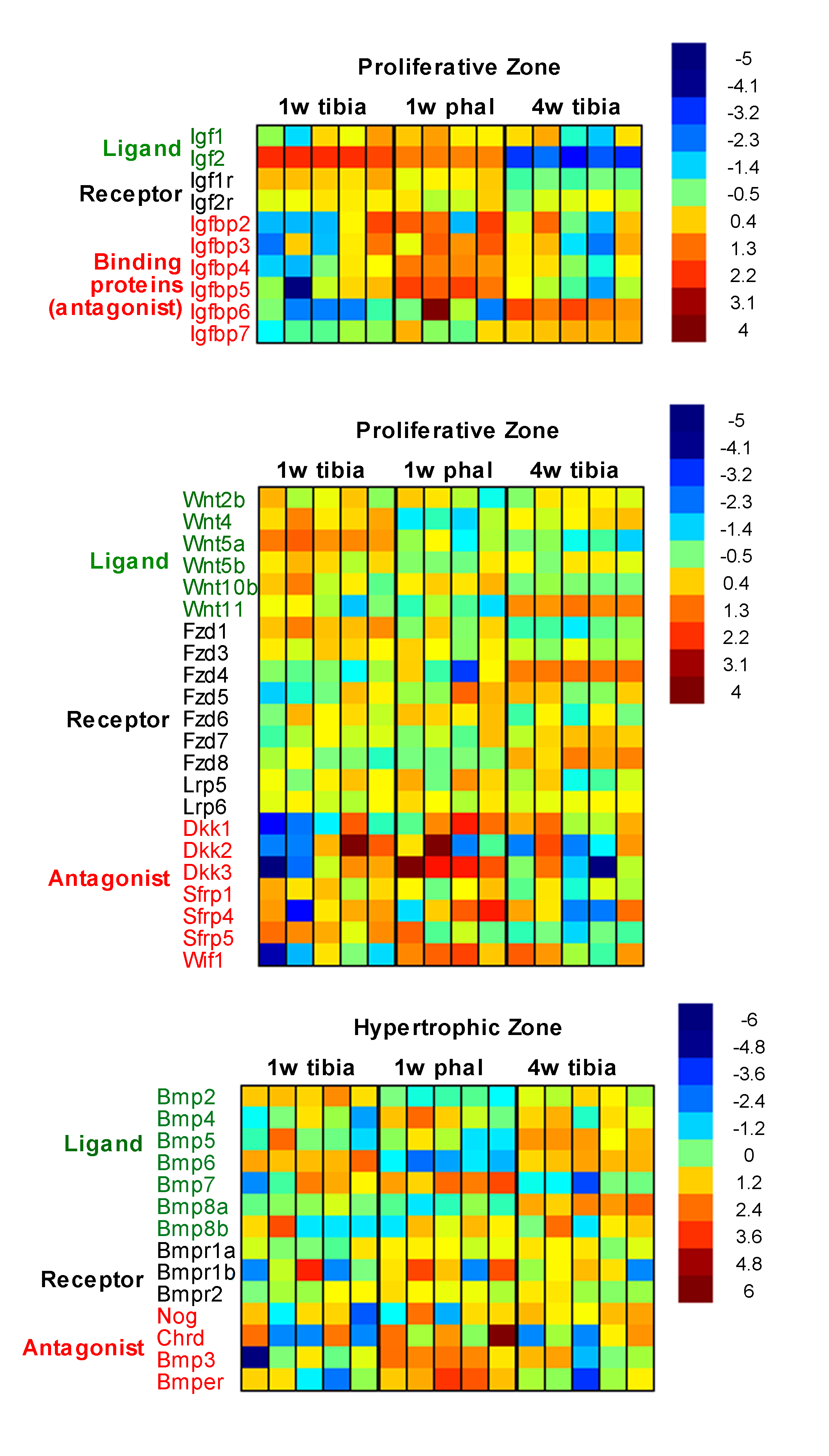

Supplement: S7 Fig — Genes were arranged by functional categories rather than by hierarchical clustering. Ligands, green; receptors, black; functional antagonists, red. Scale bar represents log2 (fold differences). Raw values used to generate the heatmaps are available in S1 Data. BMP, bone morphogenetic protein; IGF, insulin-like growth factor; RNA-Seq, RNA sequencing; WNT, Wingless and Int-1. (TIF) [file pbio.2005263.s007.tif]

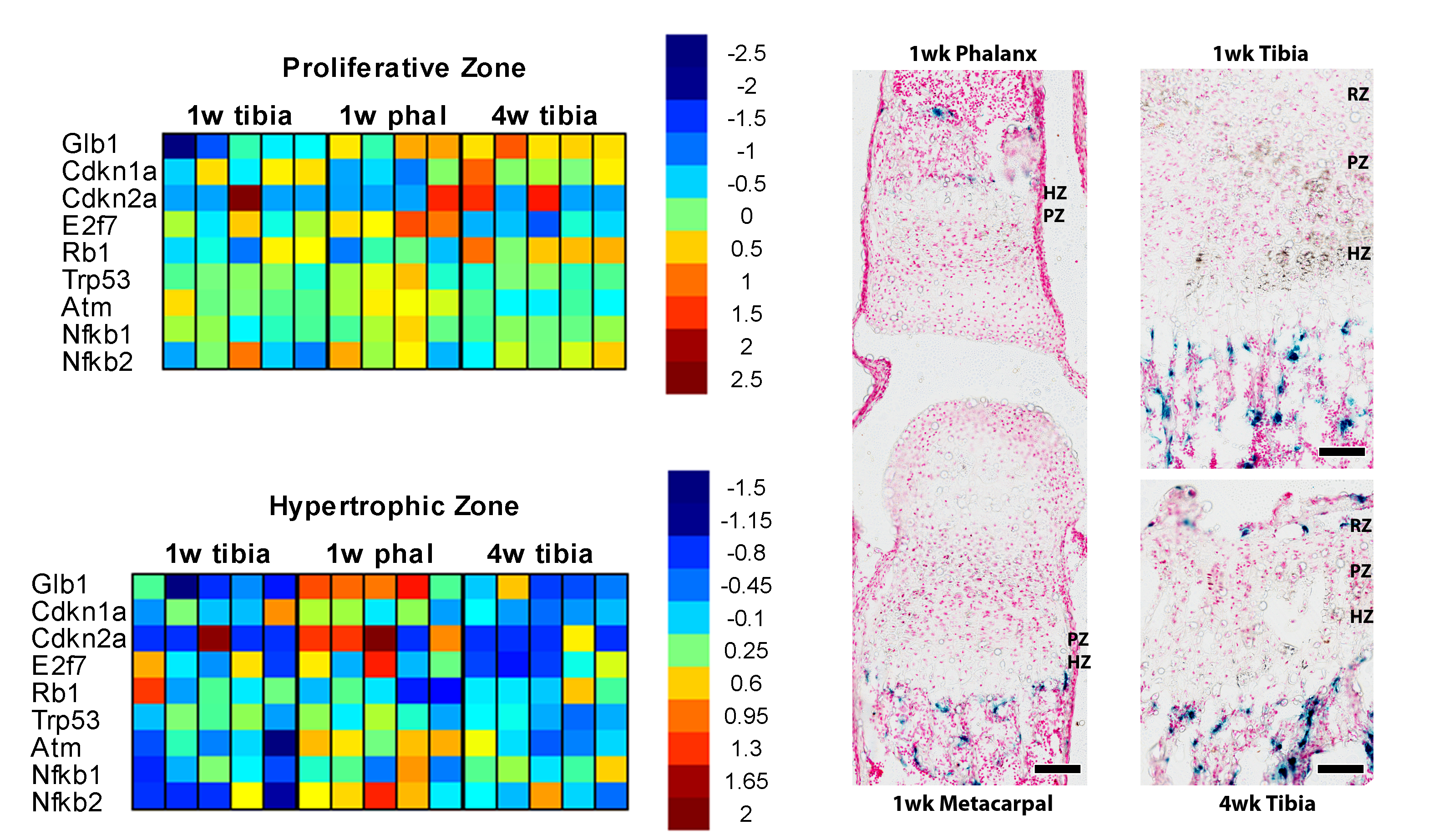

Supplement: S8 Fig — Left panels: markers of cellular senescence (genes that are known to show increased expression in senescent cells) were analyzed by RNA-Seq in proliferative and hypertrophic zones of 1- and 4-week tibia and 1-week phalanx. Scale bar represents log2 (fold differences). Raw values used to generate the heatmaps are available in S1 Data. Right panels: senescence-associated beta-galactosidase, which is a widely used marker for cellular senescence, was examined by X-gal staining in freshly frozen sections of 1- and 4-week tibias and 1-week metacarpal/phalanges. Scale bar, 100 μm. RNA-Seq, RNA sequencing. (TIF) [file pbio.2005263.s008.tif]
